# Supplementary material for: Real-time synthesis of imagined speech processes from minimally invasive recordings of neural activity
Source: Commun Biol. 2021 Sep 23;4:1055. doi: 10.1038/s42003-021-02578-0 (PMC8460739; doi:10.1038/s42003-021-02578-0)
Supplement: Supplementary file 2 — Description of Additional Supplementary Files [file 42003_2021_2578_MOESM2_ESM.pdf]

Description of Additional Supplementary Files

File Name: Supplementary Video 1

Description: This study is accompanied by a video of the patient during the experiment, which showcases examples of synthesized audio for all three communication modalities. For each modality, we present the ongoing high-gamma activity as changing color intensities at the corresponding electrodes in a brain model, in conjunction with the decoded spectrogram and waveform results, synchronized with the synthesized audio playback. The target word for each trial is indicated as the label on the x-Axis of the timeline
